# Inorganic Arsenic and Basal Cell Carcinoma in Areas of Hungary, Romania, and Slovakia: A Case–Control Study

**DOI:** 10.1289/ehp.1103534

**Published:** 2012-01-31

**Authors:** Giovanni Leonardi, Marie Vahter, Felicity Clemens, Walter Goessler, Eugen Gurzau, Kari Hemminki, Rupert Hough, Kvetoslava Koppova, Rajiv Kumar, Peter Rudnai, Simona Surdu, Tony Fletcher

**Affiliations:** 1 Centre for Radiation, Chemical and Environmental Hazards, Health Protection Agency, Chilton, United Kingdom; 2 Department of Social and Environmental Health Research, Public Health, London School of Hygiene and Tropical Medicine, London, United Kingdom; 3 Institute of Environmental Medicine, Karolinska Institutet, Stockholm, Sweden; 4 Department of Medical Statistics, Epidemiology and Population Health, London School of Hygiene and Tropical Medicine, London, United Kingdom; 5 Institut für Chemie—Analytische Chemie, Karl-Franzens-Universität, Graz, Austria; 6 Health Department, Environmental Health Center, Babes Bolyai University, Cluj-Napoca, Romania; 7 Division of Molecular Genetic Epidemiology, German Cancer Research Center, Heidelberg, Germany; 8 Soils Group, James Hutton Institute, Aberdeen, Scotland, United Kingdom; 9 Department of Environmental Health, Regional Authority of Public Health, Banska Bystrica, Slovakia; 10 Department of Environmental Epidemiology, National Institute of Environmental Health, Budapest, Hungary; 11 School of Public Health, University at Albany, State University of New York, Albany, New York, USA

**Keywords:** low-dose arsenic, metabolism, methylation, skin neoplasms, urine

## Abstract

Background: Inorganic arsenic (iAs) is a potent carcinogen, but there is a lack of information about cancer risk for concentrations < 100 μg/L in drinking water.

Objectives: We aimed to quantify skin cancer relative risks in relation to iAs exposure < 100 μg/L and the modifying effects of iAs metabolism.

Methods: The Arsenic Health Risk Assessment and Molecular Epidemiology (ASHRAM) study, a case–control study, was conducted in areas of Hungary, Romania, and Slovakia with reported presence of iAs in groundwater. Consecutively diagnosed cases of basal cell carcinoma (BCC) of the skin were histologically confirmed; controls were general surgery, orthopedic, and trauma patients who were frequency matched to cases by age, sex, and area of residence. Exposure indices were constructed based on information on iAs intake over the lifetime of participants. iAs metabolism status was classified based on urinary concentrations of methylarsonic acid (MA) and dimethylarsinic acid (DMA). Associations were estimated by multivariable logistic regression.

Results: A total of 529 cases with BCC and 540 controls were recruited for the study. BCC was positively associated with three indices of iAs exposure: peak daily iAs dose rate, cumulative iAs dose, and lifetime average water iAs concentration. The adjusted odds ratio per 10-μg/L increase in average lifetime water iAs concentration was 1.18 (95% confidence interval: 1.08, 1.28). The estimated effect of iAs on cancer was stronger in participants with urinary markers indicating incomplete metabolism of iAs: higher percentage of MA in urine or a lower percentage of DMA.

Conclusion: We found a positive association between BCC and exposure to iAs through drinking water with concentrations < 100 μg/L.

Inorganic arsenic (iAs) is a recognized carcinogen and toxicant [National Research Council (NRC) 2001; World Health Organization (WHO) International Programme on Chemical Safety 2001] that is commonly present in groundwater. Causal relationships between long-term elevated iAs exposure and cancer of the skin, lung, and bladder have been accepted [International Agency for Research on Cancer (IARC) 2004, 2009]. Although certain populations, such as in Bangladesh, West Bengal, Taiwan, parts of China, Argentina, and northern Chile, have been exposed to very high concentrations of iAs in drinking water (several hundred micrograms per liter) (IARC 2004), there is widespread exposure worldwide to low concentrations of iAs, in the range of 5–50 μg/L in drinking water and 5–100 μg/kg in food, especially cereals and vegetables (European Food Safety Authority 2009; Norton et al. 2010). The NRC risk assessment (NRC 2001) indicates a comparatively high cancer risk even at concentrations as low as 10 μg/L in drinking water; however, these risk estimates, as well as current WHO, European Union, and U.S. drinking water guidelines (European Council 1998; U.S. Environmental Protection Agency 2001; WHO 2011), are based on linear extrapolation of cancer risks at low doses in studies with relatively high iAs exposure, mainly in Taiwan (Chen et al. 1985, 1992; Chiou et al. 2001; Tseng et al. 1968; Wu et al. 1989). Concerns have been raised about the validity of such extrapolation, in part because accepted modes of action, which do not include direct DNA mutations, would be expected to result in a threshold dose response (Snow et al. 2005).

The effects of elevated iAs exposure on the risk of nonmelanoma skin cancer, mainly squamous cell carcinoma and basal cell carcinoma (BCC), have been recognized in highly exposed populations for some time (Cabrera and Gomez 2003; Chen and Wang 1990; Chen et al. 1985, 2003; Guha Mazumder et al. 1998; Guo et al. 1998; Hsueh et al. 1995; Knobeloch et al. 2006; Tsai et al. 1999; Tseng 1977; Tseng et al. 1968). However, there is little direct evidence of skin cancer risk resulting from exposure to drinking water containing < 100 μg/L iAs (Baastrup et al. 2008; Karagas et al. 2001).

The Arsenic Health Risk Assessment and Molecular Epidemiology (ASHRAM) study aimed to quantify the risks of several cancer types in relation to long-term low-level iAs exposure via drinking water in Hungary, Romania, and Slovakia. The associations with BCC of the skin are presented here.

## Materials and Methods

For the ASHRAM study, the study areas included districts in central Slovakia where drinking water is derived from a cracked hard rock aquifer, as well as districts in eastern Hungary and western Romania located on the Great Hungarian Plain, an alluvial basin that straddles the Hungarian–Romanian border. More precisely, the study included the Hungarian counties Bács-Kiskun, Békés, Csongrád, and Jász-Nagykun-Szolnok; the Romanian counties Arad and Bihor; and the Slovakian districts Nitra, Nove Zamky, and Levice within Nitra County, and Banska Bystrica, Brezno, Ziar nad Hronom, and Zarnovica within Banska Bystrica County (total population ~ 4.5 million). Routine monitoring of sources indicated that approximately 1,100,000 individuals had used water with > 10 μg iAs/L, but generally < 100 μg/L, at some point during the past 30 years (Hough et al. 2010). The study was conducted with individuals who provided written informed consent and were part of the majority Caucasian population, excluding the minority Roma population. Ethical approval was obtained from the Ethical Committee of the National Health Research Council and the Regional Ethical Committee of the Szentgyörgyi Albert University of Szeged (Hungary), from local hospitals and public health departments (Romania), and from ethical committees established in hospitals and state health institutes (Slovakia) included in the study.

In the absence of population registers, we worked with public health agencies to identify incident cases of BCC [*International Classification of Diseases, 10th Revision* (ICD-10) code C44 (WHO 1993)] and controls diagnosed among those 30–79 years of age in the same areas. Recruitment of cases and controls was carried out over 21 months (January 2003–September 2004). Because BCC cases may be diagnosed in primary or private care, a case–control study of BCC based on cases identified only at hospitals may provide an incomplete ascertainment. However, we determined that pathologists at public hospitals were responsible for histological confirmation of all BCC cases in the study areas. Therefore, identification of BCC cases entailed a system whereby each pathologist would inform the local study coordinator every time a new case of BCC was diagnosed by histology, so that the identification of BCC cases did not require cooperation by the large network of hospital- and community-based clinicians responsible for diagnosing cases.

Hospital-based case–control studies have been criticized because of the potential for selection bias resulting from recruitment of controls from a population systematically different from the source population for cases. In addition, selection bias would result if recruitment of cases and controls differed by geographical area that may be associated with degree of potential iAs exposure. Therefore, to ensure equivalent geographic coverage of populations for sampling both cases and controls, we recruited controls from all 24 hospitals in the study area, including six smaller hospitals in more peripheral areas that did not have pathology departments involved in the histological confirmation of BCC cases. All of the targeted hospitals agreed to participate in the study. Controls were general surgery in-patients (appendicitis, abdominal hernia, duodenal ulcer, or cholelithiasis, with ICD-10 diagnostic codes K35–K37, K40–K46, K26, K80) and orthopedic and trauma patients (fractures, with ICD-10 diagnostic codes SO2, S12, S22, S32, S42, S52, S62, S72, S82, S92, TO2, TO8, T10, T12) 30–79 years of age. We recruited controls diagnosed with a variety of conditions from two distinct hospital departments to reduce the possibility that geographic variation in patterns of diagnosis and clinical practice might lead to systematic differences between control and base population distributions.

Controls served as the comparison group for analyses of other cancers (bladder and kidney) in addition to BCC. Therefore, controls were frequency matched to all potential cancer cases by sex, 5-year age band, and residence in the same county/region of the study area. Cases and controls were included if they had resided in the study area for at least 1 year during their lifetime. Because a complete roster of all eligible controls admitted to all the hospitals in the region was not available, recruitment of controls was proportional to the expected number of potential controls at each hospital indicated by past data. Control selection continued until the target number of interviews was completed. This procedure led to a systematic rotation between hospitals and control diagnoses and therefore constructed a series of controls that was similar to that of the cases for age group, sex, and county of residence while minimizing the opportunity for systematic error in control selection (Leonardi et al. 2004). Clinicians and pathologists were blind to the exposure status of cases and controls.

A face-to-face interview was conducted with each participant to obtain a detailed residential history focused on identification of drinking water sources at home and information on potential confounders, including sunlight exposure, skin characteristics, educational attainment, sex, age, smoking history, height, and weight reported for age 20, before their illnesses. Participants also completed interviewer-administered food frequency questionnaires (Hough 2010). Interviewers from the three countries were staff of public health institutes who had attended training workshops to ensure consistent approach to data collection following the study protocol. Interviewers asked participants about the hours per day spent in the sun at different ages over their lifetime, as well as self-reported skin complexion, eye color, skin reaction to midday sun, and history of sunburns and red skin from sun exposure. From the questionnaire responses, four indices of exposure to sunlight were generated: peak exposure was defined as the highest annual number of hours of exposure to the sun; cumulative lifetime exposure, as the lifetime number of hours of exposure to the sun; skin sensitivity to burns, as an index based on the intensity of the cutaneous reaction to 1-hr midday sun exposure of the upper trunk; and skin complexion, as the self-reported complexion of the skin as light, medium, or dark.

*Exposure model.* For each individual, the concentrations of iAs in drinking water at addresses over their lifetime were derived from measurements at the time of the study and historical data provided by national water authorities as described in detail elsewhere (Hough et al. 2010). When historical data were unavailable, past concentrations of iAs in village and private wells were assumed to be the same as currently measured concentrations in water samples from the same wells. Address and water source data were collected on up to nine different residences over each person’s lifetime, and, at minimum, we attempted to measure iAs in current samples obtained from the first, last, and longest reported addresses. All water samples were analyzed by the same laboratory in Cluj-Napoca, Romania, using hydride generation/atomic absorption spectrometry accompanied by strict quality assurance and quality control (QA/QC) procedures. Based on sample measurements and historical information, concentrations of iAs in residential supplies were assigned for 80.09% of case and 80.07% of control lifetime years. Based on these lifetime concentration profiles and individual fluid intakes, three exposure indices were constructed: the peak daily dose rate (micrograms iAs per day) at the participant’s residence with the highest water iAs concentration, the time-weighted average concentration (micrograms iAs per liter) over the lifetime of each individual, and the lifetime cumulative dose (grams iAs) (Hough et al. 2010).

Each participant was asked to provide an early morning urine sample. The samples were placed in a freezer at –20°C within 15 min of collection to limit influence on the chemical species of iAs in urine. Urinary metabolites of iAs, methylarsonic acid [MA, MA(V) unless otherwise noted] and dimethylarsinic acid [DMA, DMA(V) unless otherwise noted] were measured with high-performance liquid chromatography with inductively coupled plasma/mass spectrometry (Lindberg et al. 2006). Total urinary metabolites were computed as the sum of iAs, MA, and DMA. Concentrations of iAs in urine were adjusted to the average specific gravity in the population. Details on concentrations of iAs in water and urine samples and their relevance for exposure assessment are available elsewhere (Lindberg et al. 2006).

*Statistical analyses.* Data were analyzed in STATA (version 10; StataCorp 2007) using separate multivariable logistic regression models for each iAs exposure index that included variables representing potential confounders defined *a priori*: several indices of sun exposure, age, sex, number of years of education (proxy for socioeconomic status), and county. Preliminary analyses identified skin response to 1-hr midday sun and skin complexion as the sunlight exposure indices most strongly predicting BCC risk in this population, so these were included in models including exposure. This population was part of a larger study of skin, bladder, and kidney cancers with a common referent population, and the targets were for similar numbers of controls and all cancers per county. The relative numbers of the different cancers varied geographically, so the BCC case:control ratio varied arbitrarily by county (and country). This geographic matching was accounted for in analyses by using logistic regression conditional on county (STATA command xtlogit, fe), which also controlled for variation across counties (clustering) of unmeasured risk factors.

Quintiles of exposure for the controls were defined for each exposure index and used for classifying cases and controls to explore the shape of the relationships. The trend across quintile was assessed by fitting a linear term for exposure quintile. To evaluate possible departure from linearity, likelihood ratio tests were used to compare the fit of models with exposure quintile as a categorical variable versus quintile as a linear term.

Each index was also treated as a continuous variable in regression models to estimate the overall slope of the exposure response, with skin cancer relative risk expressed as odds ratios (ORs) for unit changes in each exposure: 10-μg/L increase in lifetime average iAs concentration, 10-μg/day increase in peak iAs daily dose rate, and increase of 0.274 g in cumulative iAs dose derived from 50 years of drinking 1.5 L water per day containing 10 μg/L iAs.

We conducted analyses adjusted for smoking and body mass index (BMI), but including these covariates did not alter the magnitude of associations very much. Because neither smoking nor BMI is a likely confounder in a study of BCC, we did not include them in the reported analyses.

Because other iAs exposure routes, particularly diet, may be significant when concentrations of iAs in drinking water are low (Lindberg et al. 2006), we performed analyses that excluded those with a sum of iAs metabolites in urine < 2.5 μg/L (in line with Lindberg et al. 2006). Because of potential interest in exposure to < 50 μg/L of iAs in drinking water, a further sensitivity analysis was carried out after excluding participants with lifetime average iAs concentrations above the 90th percentile of lifetime average iAs concentrations (40.7 μg/L), within the group with a sum of iAs metabolites ≥ 2.5 μg/L.

The principal metabolic data were *a priori* defined as the percentage of MA and DMA (in relation to total concentration of iAs metabolites) in the urine (MA% and DMA%, respectively). Stratified analyses were conducted to evaluate effect modification by methylation efficiency on the association between BCC and iAs exposure. Two dichotomous variables representing DMA% and MA% below and above the median were modeled along with multiplicative interaction terms between each dichotomous variable and the iAs exposure indices (as continuous variables). Modification of the association between iAs and BCC by DMA and MA was evaluated based on Wald tests (Clayton and Hills 1993).

## Results

Of the 1,406 patients invited, 1,197 (85.1%) consented to participate in the study (81.6% among cases, 90.0% among controls). Later, 96 potential cases were excluded because they had skin cancer other than BCC, 34 because they did not have histological confirmation, and 4 for other reasons. Thus, in total, the study included 1,069 valid participants, of whom 529 were BCC cases and 540 were controls. Consistent with expectations for BCC, a cancer known to be caused by sun exposure, cases had a higher prevalence of skin burns in response to sun exposure than did controls, as well as a higher prevalence of light skin complexion (Table 1).

**Table 1:** Study population and characteristics [n (%)].

Characteristic	Cases	Controls	Total (n)
Country/county						
Hungary, total		160 (30.3)		249 (46.1)		409
Bács-Kiskun		90 (17.1)		101 (18.7)		191
Békés		23 (4.4)		23 (4.3)		46
Csongrád		24 (4.5)		61 (11.3)		85
Jász-Nagykun-Szolnok		23 (4.4)		64 (11.9)		87
Romania, total		158 (29.9)		156 (28.9)		314
Arad		90 (17.0)		100 (18.5)		190
Bihor		68 (12.9)		56 (10.4)		124
Slovakia, total		211 (39.9)		135 (25.0)		346
Banska Bystrica		92 (17.4)		70 (13.0)		162
Nitra		119 (22.5)		65 (12.0)		184
Age (years)						
< 45		20 (3.9)		55 (10.3)		75
45–49		33 (6.4)		47 (8.8)		80
50–54		31 (6.0)		61 (11.4)		92
55–59		63 (12.1)		77 (14.5)		140
60–64		65 (12.5)		76 (14.3)		141
65–69		106 (20.4)		83 (15.6)		189
70–74		113 (21.7)		78 (14.7)		191
75–79		89 (17.1)		56 (10.5)		145
Missing		9		7		16
Sex						
Male		237 (44.8)		278 (51.5)		515
Female		292 (55.2)		262 (48.5)		554
Skin response to 1-hr midday sun						
Blistered		71 (13.7)		59 (11.1)		130
Sunburned		114 (22.0)		83 (15.6)		197
Mild burn		169 (32.6)		161 (30.3)		330
Tan		157 (30.3)		206 (38.8)		363
No change		7 (1.4)		22 (4.1)		29
Missing		11		9		20
Skin complexion						
Light		271 (51.3)		222 (41.2)		493
Medium		241 (45.6)		258 (47.9)		499
Dark		16 (3.0)		59 (11.0)		75
Missing		1		1		2
Total		529 (100.0)		540 (100.0)		1,069
Years of education (mean ± SD)		10.1 ± 4.1		10.2 ± 3.7		10.1 ± 3.9

The lifetime average iAs concentration in residential drinking water had a median value of 1.2 μg/L, with an interquartile range (IQR) of 0.7–13.8 μg/L. It is evident from the distribution of lifetime average iAs concentration (quintiles of controls) shown in Table 2 that much of the population (first three quintiles) had exposure < 7 μg/L.

**Table 2:** Results of logistic regression models of iAs exposure by quintile and BCC in the ASHRAM study population [OR (95% confidence interval)].

Arsenic exposure index/quintile (range of exposure in controls)	Adjusteda	Additionally adjustedb	Trend test (p-value)
Lifetime average iAs concentration (μg/L)						0.001
0.00–0.68		1.00		1.00		
0.68–0.98		1.27 (0.82, 1.97)		1.39 (0.89, 2.19)		
0.98–7.00		1.02 (0.67, 1.56)		1.20 (0.77, 1.88)		
7.10–19.43		1.63 (0.93, 2.85)		1.73 (0.97, 3.11)		
19.54–167.29		2.81 (1.62, 4.87)		3.03 (1.70, 5.41)		
Peak daily iAs dose rate (μg/day)						0.001
0.00–0.73		1.00		1.00		
0.73–1.48		0.93 (0.62, 1.39)		0.91 (0.59, 1.39)		
1.48–9.09		1.29 (0.86, 1.95)		1.55 (1.00, 2.41)		
9.09–32.23		1.78 (1.05, 3.02)		1.76 (1.01, 3.07)		
32.23–242.14		2.31 (1.32, 4.03)		2.50 (1.39, 4.49)		
Cumulative iAs dose (g)						0.001
0.00–0.01		1.00		1.00		
0.01–0.03		1.02 (0.68, 1.52)		1.09 (0.72, 1.67)		
0.03–0.13		1.19 (0.78, 1.81)		1.46 (0.93, 2.27)		
0.13–0.55		1.73 (1.02, 2.91)		1.76 (1.02, 3.04)		
0.55–4.46		2.45 (1.39, 4.32)		2.63 (1.45, 4.78)		
Relative risks of BCC were estimated as ORs comparing risk of cancer in a quantile with the quintile of lowest exposure. Range of iAs exposure within each quintile is expressed in the unit of measure specific for each exposure index. aAdjusted for county, age, and sex, based on n = 1,022 for peak dose rate, n = 1,025 for lifetime average concentration, and n = 1,011 for cumulative dose. bAdditionally adjusted for education, skin response to 1-hr midday sun, and skin complexion, based on n = 989 for peak dose rate, n = 992 for lifetime average concentration, n = 979 for cumulative dose.						

*Arsenic and risk of BCC.* In general, ORs increased with increasing iAs exposure categories, and all trend tests for the linear relationship between the categorical exposure variable and BCC were statistically significant (Table 2). Comparison by likelihood ratio tests of models with quintile of exposure as a categorical variable and a linear variable did not provide evidence of nonlinearity in the exposure–response relationship. Subsequent analyses were conducted based on the assumption that the dose–response relation between iAs exposure indexes and BCC can be summarized appropriately using simple continuous exposure variables.

All three iAs exposure indices were significantly associated with BCC when adjusted for county, age, and sex, and ORs were comparable after further adjustment for education, skin response to 1-hr midday sun, and skin complexion (Table 2). Associations were also significant when exposures were modeled as continuous variables, with similar effect estimates after excluding individuals whose sum of iAs metabolites in current urine was < 2.5 μg/L (Table 3). Associations were increased after further excluding participants with lifetime average iAs water concentration above the 90th percentile (41 μg/L).

**Table 3:** Logistic regression models of iAs exposure and BCC in the ASHRAM study population [OR (95% confidence interval)].

Arsenic exposure index	All observations (n = 1,069)	Observations with metabolites ≥ 2.5 μg/L (n = 856)	Observations with metabolites ≥ 2.5 μg/L and lifetime average As < 40.7 μg/L (n = 736)
Lifetime average As concentration		1.18 (1.08, 1.28)		1.15 (1.06, 1.26)		1.41 (1.14, 1.76)
Peak daily As dose rate		1.13 (1.07, 1.20)		1.12 (1.06, 1.19)		1.18 (1.04, 1.33)
Cumulative As dose		1.10 (1.01, 1.19)		1.08 (1.00, 1.18)		1.16 (0.95, 1.41)
Relative risks of BCC were estimated as ORs for unit changes in exposure: a 10-μg/L increase in lifetime average iAs concentration, a 10‑μg/day increase in peak iAs daily dose rate, or an increase in cumulative iAs dose derived from 50 years of drinking 1.5 L water/day containing 10 μg/L iAs. All models adjusted for county, age, sex, education, skin response to 1-hr midday sun, and skin complexion.						

*Effect modification by iAs metabolism.* Median DMA% was 76.6% (median concentration, 4.0 μg/L; IQR, 2.1–9.7 μg/L), and median MA% was 15.8% (median concentration, 0.8 μg/L IQR, 0.4–2.1 μg/L; Table 4). Among participants with sum of iAs metabolites ≥ 2.5 μg/L, associations with all three indices of iAs exposure were stronger in participants with low DMA% or high MA%, with little or no evidence of associations among participants with high DMA% or low MA%. Similar results were obtained for the overall group (including those with metabolites < 2.5 μg/L; data not shown).

**Table 4:** Associations between BCC and As according to urine iAs metabolite levels among participants with sum of iAs metabolites ≥ 2.5 μg/L (n = 856).

Arsenic exposure index	< Mediana [ORb (95% CI)]	≥ Mediana [ORb (95% CI)]	p-Valuec
Lifetime average concentration						
DMA%		1.21 (1.10, 1.35)		1.03 (0.92, 1.15)		0.017
MA%		1.04 (0.94, 1.17)		1.21 (1.09, 1.35)		0.032
Peak daily As dose rate						
DMA%		1.14 (1.06, 1.23)		1.05 (0.96, 1.15)		0.098
MA%		1.05 (0.97, 1.15)		1.15 (1.06, 1.23)		0.095
Cumulative As dose						
DMA%		1.11 (1.01, 1.22)		0.99 (0.88, 1.12)		0.119
MA%		1.02 (0.91, 1.14)		1.10 (1.00, 1.22)		0.222
Relative risks of BCC were estimated as ORs for unit changes in exposure: a 10 μg/L increase in lifetime average iAs concentration, a 10-μg/day increase in peak iAs daily dose rate, or an increase in cumulative iAs dose derived from 50 years of drinking 1.5 L water/day containing 10 μg/L iAs. aMedian DMA%, 76.6%; median MA%, 15.8%. bThese ORs are stratum specific because they estimate effect of iAs on cancer as a linear effect, separately by level of metabolite, for both DMA% and MA%, and controlled for potential confounders county, age, sex, education, skin response to 1-hr midday sun, and skin complexion. cp-Values for multiplicative interaction terms between dichotomous variables for DMA% or MA% and continuous variables for iAs exposure indexes (Wald test).						

## Discussion

The ASHRAM study demonstrates strong evidence of an association between long-term low-level exposure to iAs in drinking water and BCC. To our knowledge this is the first report of a significant association between BCC and average drinking water iAs concentrations < 50 μg/L—concentrations common in many countries and affecting many millions of people. In addition, individuals with less efficient iAs methylation to DMA appeared to be at higher risk of BCC in association with iAs exposure. The relative risk of BCC was elevated in relation to all three indices of long-term iAs exposure (lifetime average concentration, cumulative and peak dose), and the relationship remained significant after controlling for potential confounders, including indices of ultraviolet exposure.

Hospital-based case–control studies with a specific disease outcome have as their secondary population base a cohort of people who theoretically would be admitted to that hospital had they contracted the outcome disease (Miettinen 1985; Wacholder et al. 1992a, 1992b). Selection bias could arise from systematic differences in selection of cases and controls. Difference in unmeasured confounders between countries and counties was addressed in part by including county indicator variables in all models. Geographic variation in case versus control ascertainment could potentially lead to biased estimates of iAs effect if associated with geographic variation in iAs exposure. To optimize case ascertainment, we collaborated with all pathologists qualified to make diagnoses of BCC in the study areas. To minimize the potential for selection bias associated with control selection, we included all health care facilities where control diagnoses were produced, whether or not BCC cases were diagnosed at the facility. In addition, we identified several potentially suitable control diagnoses, including both general surgery and trauma. Overall, we believe that we achieved similar countywide coverage for cases and controls and minimized potential bias due to geographic differences in ascertainment related to iAs exposure.

A validation study was conducted to compare the proportion of controls from larger cities and towns with the proportion of base population from the same towns. Together, larger cities and towns constitute a more urban “subcounty area” than the smaller towns and villages that account for the remaining portions of the study counties. From census figures in the three countries (Hungarian Central Statistical Office 2011; National Institute of Statistics, Romania 2011a, 2011b; Statistical Office of the Slovak Republic 2011), we identified base population counts for geographic areas at subcounty level (3,503,581) and in the larger cities and towns (1,238,984). The proportion of the base population in the larger cities and towns (35.4%) was comparable to the proportion of controls from the same areas (187 of 538 total controls, 34.8%), which suggests that the control group is representative of the base population. In addition, response rates were high and similar between cases and controls. However, iAs exposures were higher in the rural areas than in the urban subcounty areas, with average lifetime iAs concentrations of 2.9 μg/L and 0.9 μg/L, respectively, and some residual bias cannot be ruled out completely.

Analysis of total concentrations of iAs in drinking water in the present study was based on established analytical chemistry techniques, and the QA/QC program demonstrated that measured concentrations of iAs were within acceptable margins of error. Thus, analytical data were suitable for estimating current exposure from drinking water for all participants. Historic data provided by the water authorities were based on older (and potentially less precise) methods, combined with estimated dates when water treatments were introduced. However, these data were provided by experts blind to whether the named communities hosted cases or controls.

Information on residences over the lifetime of participants, which was used to identify residences where water sources were available for sampling or to link past residences with historical iAs measurement data, was complete for most of the participants’ life-years (80%). The reconstruction of historic exposure to iAs in drinking water can therefore be considered adequate for production of exposure assessment estimates and provides a more complete characterization of overall exposure than from current sources alone (Hough et al. 2010).

Most of the ASHRAM study population was using drinking water with low iAs concentrations (75% < 14 μg/L), well below concentrations in studies upon which the current drinking water standard is based. The use of individual measurements of iAs in drinking water for exposure estimation was validated by comparing the concentrations of iAs in the currently used water with the concentrations of iAs metabolites in urine, which is considered a good indicator of ongoing exposure (Lindberg et al. 2006). The concentrations of iAs in urine were significantly correlated with those in water (*R*^2^ = 0.46; *p* < 0.001).

Several potential confounders were measured and included in the analyses and had minimal effect. However, incomplete adjustment for confounders remains a possibility.

We estimated the iAs effect when the analyses were restricted to the subgroup with sum of iAs metabolites ≥ 2.5 μg/L, based on the assumption that the relative contribution of food iAs intake to overall exposure is relatively low in this group. We found that associations between BCC and iAs exposure via water were very similar between this subgroup and the overall study population.

The possibility that exposure to low concentrations of iAs may be associated with BCC has been debated (Brown and Ross 2002; Schoen et al. 2004). To exclude the possibility that the association between BCC and iAs was mainly due to the few cases using water with relatively high iAs concentration, we repeated the analyses after excluding cases and controls with an average iAs concentration at or above the 90th percentile of 40.7 μg/L. Associations within this subgroup were similar to, or somewhat stronger than, those for the overall study population, which suggests that if there is a threshold for iAs effects, it may be below an iAs lifetime average concentration of 40.7 μg/L. There is increasing evidence based on mechanistic research that very low concentrations of iAs induce cell proliferation, telomerase expression, increased telomere length, and oncogene overexpression, whereas higher concentrations induce apoptosis, telomerase inhibition, and decreased telomere length (Ferrario et al. 2009; Hwang et al. 2006; Mo et al. 2009; Vega et al. 2001). There is additional mechanistic support for effects of iAs at very low dose levels, for example, via epigenetic and endocrine interactions (Arita and Costa 2009; Davey et al. 2007; Singh and DuMond 2007), as well as inhibition of DNA repair (Nollen et al. 2009). DNA repair genes may influence individual susceptibility to BCC (Thirumaran et al. 2006), and their role in iAs-related cancer also needs further investigation. In view of these possibilities, the overall pattern of iAs carcinogenic effects at low concentrations and possible mechanisms for low-dose effects need further study.

The pattern of risk in subgroups defined on the basis of iAs metabolism (DMA% and MA%) is broadly consistent with those of recent studies (Chen et al. 2003; Hsueh et al. 1997; Yu et al. 2000) and suggests increasing risk of iAs-related skin cancer with increasing percentage of MA in urine. Most of the populations previously studied experienced relatively high exposures, at which iAs methylation is inhibited, resulting in an increased percentage of urinary MA (Lindberg et al. 2007; Vahter 2002). Our findings suggest that the influence of metabolism is still present at fairly low iAs exposure levels, where iAs metabolism is mainly influenced by genetic predisposition (Lindberg et al. 2007). Possibly, MA in urine reflects the tissue levels of MA(III), a highly toxic intermediate metabolite (Drobná et al. 2005; Kligerman et al. 2003).

## Conclusions

We found positive associations between long-term exposure to iAs < 100 μg/L in drinking water and BCC of the skin. The exposure metric that showed the most significant relationship with BCC was lifetime average water iAs concentration. The association between iAs and BCC remained significant in the subgroup exposed to iAs lifetime average water iAs concentration < 40 μg/L (90th percentile). Arsenic metabolism modified the risk of BCC such that the association with iAs appeared to be limited to participants with reduced iAs methylation efficiency, reflected in low DMA% and high MA% in urine. Overall, our findings add to the evidence that low-dose exposure to iAs causes cancer and support current recommendations to minimize human exposure even at relatively low concentrations.

## References

[r1] Arita A, Costa M. (2009). Epigenetics in metal carcinogenesis: nickel, arsenic, chromium and cadmium.. Metallomics.

[r2] Baastrup R, Sorensen M, Balstrøm T, Frederiksen K, Larsen CL, Tjonneland A (2008). Arsenic in drinking-water and risk for cancer in Denmark.. Environ Health Perspect.

[r3] Brown KG, Ross GL (2002). Arsenic, Drinking Water, and Health (A Position Paper of the American Council on Science and Health)..

[r4] Cabrera HN, Gomez ML (2003). Skin cancer induced by arsenic in the water.. J Cutan Med Surg.

[r5] Chen CJ, Chen CW, Wu MM, Kuo TL (1992). Cancer potential in liver, lung, bladder and kidney due to ingested inorganic arsenic in drinking water.. Br J Cancer.

[r6] Chen CJ, Chuang YC, Lin TM, Wu HY (1985). Malignant neoplasms among residents of a blackfoot disease-endemic area in Taiwan: high-arsenic artesian well water and cancers.. Cancer Res.

[r7] Chen CJ, Wang CJ (1990). Ecological correlation between arsenic level in well water and age-adjusted mortality from malignant neoplasms.. Cancer Res.

[r8] Chen Y-C, Guo Y-LL, Su H-JJ, Smith TJ, Ryan LM, Lee M-S (2003). Arsenic methylation and skin cancer risk in Southwestern Taiwan.. J Occup Environ Med.

[r9] Chiou HY, Chiou ST, Hsu YH, Chou YL, Tseng CH, Wei ML (2001). Incidence of transitional cell carcinoma and arsenic in drinking water: a follow-up study of 8,102 residents in an arseniasis-endemic area in northeastern Taiwan.. Am J Epidemiol.

[r10] Clayton D, Hills M (1993). Statistical Models in Epidemiology..

[r11] Davey JC, Bodwell JE, Gosse JA, Hamilton JW (2007). Arsenic as an endocrine disruptor: effects of arsenic on estrogen receptor-mediated gene expression *in vivo* and in cell culture.. Toxicol Sci.

[r12] Drobná Z, Waters SB, Devesa V, Harmon AW, Thomas DJ, Styblo M (2005). Metabolism and toxicity of arsenic in human urothelial cells expressing rat arsenic (+3 oxidation state)-methyltransferase.. Toxicol Appl Pharmacol.

[r13] European Council (1998). European Council Directive 98/83/EC of 3 November 1998 on the quality of water intended for human consumption.. Official Journal of the European Communities 5.12.98..

[r14] European Food Safety Authority 2009 EFSA Panel on Contaminants in the Food Chain (CONTAM); scientific opinion on arsenic in food. EFSA J 7 10 1351; doi: [Online 12 October 2009] 10.2903/j.efsa.2009.1351

[r15] Ferrario D, Collotta A, Carfi M, Bowe G, Vahter M, Hartung T (2009). Arsenic induces telomerase expression and maintains telomere length in human cord blood cells.. Toxicology.

[r16] Guha Mazumder DN, Haque R, Ghosh N, De BK, Santra A, Chakraborty D (1998). Arsenic levels in drinking water and the prevalence of skin lesions in West Bengal, India.. Int J Epidemiol.

[r17] Guo HR, Lipsitz SR, Hu H, Monson RR (1998). Using ecological data to estimate a regression model for individual data: the association between arsenic in drinking water and incidence of skin cancer.. Environ Res.

[r18] Hough RL, Fletcher T, Leonardi GS, Goessler W, Gnagnarella P, Clemens F (2010). Lifetime exposure to arsenic in residential drinking water in Central Europe.. Int Arch Occup Environ Health.

[r19] Hsueh YM, Cheng GS, Wu MM, Yu HS, Kuo TL, Chen CJ (1995). Multiple risk factors associated with arsenic-induced skin cancer: effects of chronic liver disease and malnutritional status.. Br J Cancer.

[r20] Hsueh YM, Chiou HY, Huang YL, Wu WL, Huang CC, Yang MH (1997). Serum beta-carotene level, arsenic methylation capability, and incidence of skin cancer.. Cancer Epidemiol Biomarkers Prev.

[r21] Hungarian Central Statistical Office (2011). Population Estimation Dissemination Database.. The Continuous Registration of the Population by Settlement.

[r22] Hwang BJ, Utti C, Steinberg M (2006). Induction of cyclin D1 by submicromolar concentrations of arsenite in human epidermal keratinocytes.. Toxicol Appl Pharmacol.

[r23] IARC (International Agency for Research on Cancer) (2004). Some drinking-water disinfectants and contaminants, including arsenic. Monographs on chloramine, chloral and chloral hydrate, dichloroacetic acid, trichloroacetic acid and 3-chloro-4-(dichloromethyl)-5-hydroxy-2(5H)-furanone.. IARC Monogr Eval Carcinog Risks Hum.

[r24] IARC (International Agency for Research on Cancer) (2009). A review of human carcinogens. Part C: metals, arsenic, dusts, and fibres.. Lancet Oncol.

[r25] Karagas MR, Stukel TA, Morris JS, Tosteson TD, Weiss JE, Spencer SK (2001). Skin cancer risk in relation to toenail arsenic concentrations in a U.S. population-based case–control study.. Am J Epidemiol.

[r26] Kligerman AD, Doerr CL, Tennant AH, Harrington-Brock K, Allen JW, Winkfield E (2003). Methylated trivalent arsenicals as candidate ultimate genotoxic forms of arsenic: induction of chromosomal mutations but not gene mutations.. Environ Mol Mutagen.

[r27] Knobeloch LM, Zierold KM, Anderson HA (2006). Association of arsenic-contaminated drinking-water with prevalence of skin cancer in Wisconsin’s Fox River Valley.. J Health Popul Nutr.

[r28] Leonardi GS, Fletcher T, Koppova K, Hough R, Rudnai P, Gurzau E (2004). Selection of controls for hospital-based case-control studies using retrospective data on the geographic distribution of cases and controls. Epidemiology.

[r29] Lindberg AL, Goessler W, Gurzau E, Koppova K, Rudnai P, Kumar R (2006). Arsenic exposure in Hungary, Romania, and Slovakia.. J Environ Monit.

[r30] Lindberg AL, Kumar R, Goessler W, Thirumaran R, Gurzau E, Koppova K (2007). Metabolism of low-dose inorganic arsenic in a central European population: influence of sex and genetic polymorphisms.. Environ Health Perspect.

[r31] Miettinen OS (1985). The “case–control” study: valid selection of subjects.. J Chron Dis.

[r32] Mo J, Xia Y, Ning Z, Wade TJ, Mumford JL (2009). Elevated human telomerase reverse transcriptase gene expression in blood cells associated with chronic arsenic exposure in Inner Mongolia, China.. Environ Health Perspect.

[r33] National Institute of Statistics, Romania (2011a). Regional Statistics for Year 2007: Arad County [in Romanian].. http://www.arad.insse.ro/main.php.

[r34] National Institute of Statistics, Romania (2011b). Regional Statistics for Year 2007: Bihor County [in Romanian].. http://www.bihor.insse.ro/main.php.

[r35] Nollen M, Ebert F, Moser J, Mullenders LH, Hartwig A, Schwerdtle T (2009). Impact of arsenic on nucleotide excision repair: XPC function, protein level, and gene expression.. Mol Nutr Food Res.

[r36] Norton GJ, Islam MR, Duan G, Lei M, Zhu Y, Deacon CM (2010). Arsenic shoot-grain relationships in field grown rice cultivars.. Environ Sci Technol.

[r37] NRC (National Research Council) (2001). Arsenic in Drinking Water: 2001 Update..

[r38] Schoen A, Beck B, Sharma R, Dubé E. (2004). Arsenic toxicity at low doses: epidemiological and mode of action considerations.. Toxicol Appl Pharmacol.

[r39] Singh KP, DuMond JW (2007). Genetic and epigenetic changes induced by chronic low-dose exposure to arsenic of mouse testicular Leydig cells.. Int J Oncol.

[r40] Snow ET, Sykora P, Durham TR, Klein CB (2005). Arsenic, mode of action at biologically plausible low doses: what are the implications for low dose cancer risk?. Toxicol Appl Pharmacol.

[r41] StataCorp (2007). Stata Statistical Software: Release 10.. College Station, TX:StataCorp..

[r42] Statistical Office of the Slovak Republic (2009). RegDat Regional Statistics Database (demographic data for year 2003).. http://px-web.statistics.sk/PXWebSlovak/index_en.htm.

[r43] Thirumaran RK, Bermejo JL, Rudnai P, Gurzau E, Koppova K, Goessler W (2006). Single nucleotide polymorphisms in DNA repair genes and basal cell carcinoma of skin.. Carcinogenesis.

[r44] Tsai SM, Wang TN, Ko YC (1999). Mortality for certain diseases in areas with high levels of arsenic in drinking water.. Arch Environ Health.

[r45] Tseng WP (1977). Effects and dose–response relationships of skin cancer and blackfoot disease with arsenic.. Environ Health Perspect.

[r46] Tseng WP, Chu HM, How SW, Fong JM, Lin CS, Yeh S (1968). Prevalence of skin cancer in an endemic area of chronic arsenicism in Taiwan.. J Natl Cancer Inst.

[r47] U.S. Environmental Protection Agency (2001). National Primary Drinking Water Regulations; Arsenic and Clarifications to Compliance and New Source Contaminants Monitoring; Final Rule.. http://www.epa.gov/fedrgstr/EPA-WATER/2001/January/Day-22/w1668.htm.

[r48] Vahter M. (2002). Mechanisms of arsenic biotransformation.. Toxicology.

[r49] Vega L, Styblo M, Patterson R, Cullen W, Wang C, Germolec D. (2001). Differential effects of trivalent and pentavalent arsenicals on cell proliferation and cytokine secretion in normal human epidermal keratinocytes.. Toxicol Appl Pharmacol.

[r50] Wacholder S, Silverman DT, McLaughlin JK, Mandel JS (1992a). Selection of controls in case–control studies: I. Principles.. Am J Epidemiol.

[r51] Wacholder S, Silverman DT, McLaughlin JK, Mandel JS (1992b). Selection of controls in case–control studies: III. Design options.. Am J Epidemiol.

[r52] WHO (World Health Organization) (1993). International Classification of Diseases, 10th Revision.. Geneva:WHO.

[r53] WHO (World Health Organization) (2011). Guidelines for Drinking-Water Quality. 4th ed.. http://www.who.int/water_sanitation_health/publications/2011/dwq_chapters/en/index.html.

[r54] WHO (World Health Organization) International Programme on Chemical Safety (2001). Arsenic and Arsenic Compounds.. Environmental Health Criteria 224. 2nd ed. Geneva:WHO..

[r55] Wu MM, Kuo TL, Hwang YH, Chen CJ (1989). Dose–response relation between arsenic concentration in well water and mortality from cancers and vascular diseases.. Am J Epidemiol.

[r56] Yu RC, Hsu KH, Chen CJ, Froines JR (2000). Arsenic methylation capacity and skin cancer.. Cancer Epidemiol Biomarkers Prev.

